# Therapeutic Cytokine Inhibition Modulates Activation and Homing Receptors of Peripheral Memory B Cell Subsets in Rheumatoid Arthritis Patients

**DOI:** 10.3389/fimmu.2020.572475

**Published:** 2020-09-11

**Authors:** Zafar Mahmood, Marc Schmalzing, Thomas Dörner, Hans-Peter Tony, Khalid Muhammad

**Affiliations:** ^1^Department of Medicine II, Rheumatology and Clinical Immunology, University of Würzburg, Würzburg, Germany; ^2^Department Medicine/Rheumatology and Clinical Immunology, Charité- Universitätsmedizin Berlin, DRFZ Berlin, Berlin, Germany; ^3^Department of Biology, College of Science, United Arab Emirates University, Al Ain, United Arab Emirates

**Keywords:** B cells, inflammation, adalimumab, tocilizumab (IL-6 inhibitor), memory B cells, rheumatoid arhritis

## Abstract

Memory B cells have known to play an important role in the pathogenesis of rheumatoid arthritis (RA). With the emergence of B cell-targeted therapies, the modulation of memory B cells appears to be a key therapeutic target. Human peripheral memory B cells can be distinguished based on the phenotypic expression of CD27 and IgD, characterizing the three major B cell subpopulations: CD27+IgD+ pre-switch, CD27+IgD- post-switch, and CD27-IgD- double-negative memory B cells. We evaluated different memory cell populations for activation markers (CD95 and Ki-67) and chemokine receptors (CXCR3 and 4) expressing B cells in active RA, as well as under IL6-R blockade by tocilizumab (TCZ) and TNF-α blockade by adalimumab (ADA). Memory B cells were phenotypically analyzed from RA patients at baseline, week 12, and week 24 under TCZ or ADA treatment, respectively. Using flow cytometry, surface expression of CD95, intracellular Ki-67, and surface expressions of CXCR3 and CXCR4 were determined. Compared with healthy donors (*n* = 40), the phenotypic analysis of RA patients (*n* = 80) demonstrated that all three types of memory B cells were activated in RA patients. Surface and intracellular staining of B cells showed a significantly higher percentage of CD95+ (*p* < 0.0001) and Ki-67+ (*p* < 0.0001) cells, with numerically altered CXCR3+ and CXCR4+ cells in RA. CD95 and Ki-67 expressions were highest in post-switch memory B cells, whereas CD19+CXCR3+ and CD19+CXCR4+ expressing cells were substantially higher in the pre-switch compartment. In all subsets of the memory B cells, *in vivo* IL-6R, and TNF-α blockade significantly reduced the enhanced expressions of CD95 and Ki-67. Based on our findings, we conclude that the three major peripheral memory B cell populations, pre-, post-switch, and double-negative B cells, are activated in RA, demonstrating enhanced CD95 and Ki-67 expressions, and varied expression of CXCR3 and CXCR4 chemokine receptors when compared with healthy individuals. This activation can be efficaciously modulated under cytokine inhibition *in vivo*.

## Introduction

Rheumatoid arthritis (RA) is an inflammatory systemic autoimmune disease characterized by polyarthritis with swelling, pain, inflammation, and progressing destruction of joints. It affects approximately 1% of the population worldwide. RA causes considerable morbidity, diminishes the life quality, and increases mortality with increasing age (1, 2). RA is influenced by environmental and genetic factors, with obesity, diet, smoking, and microbial infections are known to induce RA in genetically susceptible individuals (3, 4). Inflammation of joints is the hallmark of RA, comprising a syndrome of pain, stiffness, and symmetrical synovitis of diarthrodial joints. Furthermore, systemic inflammation targets other organs, with substantial comorbidities observed in neurological, cardiovascular, and metabolic systems in this inflammatory joint disease (5, 6).

The key role of B cells has been appreciated since the discovery of the rheumatoid factor (RF). Along with anti-cyclic-citrullinated peptide autoantibodies, the RF serves as a disease, as well as severity biomarker (7). Patients with RA show a heterogeneous modulation of the B cell compartment, particularly with an increased frequency of memory B cells (8, 9). Enhanced B cell activity has been proposed in the pathogenesis of RA, along with different pro-inflammatory cytokines such as interleukin 6 (IL-6) and tumor necrosis factor-alpha (TNF-α) critically involved in chronic inflammation. With a growing understanding of mechanistic pathways regarding B cell involvement in self-destruction during autoimmune diseases, there is strong evidence suggesting that B cells play a central role in the pathogenesis of several autoimmune diseases (10–12). Reportedly, a murine model of multiple sclerosis has reported that IL-6 producing B cells enhance T cell stimulation, including IL-17 polarization (13). IL-6 is a frontier cytokine in the induction of inflammation and generation of acute phase reactions, as well as regulation of immune responses. It is a multifunctional cytokine acting as a stimulator of B cells and was formerly described as a late-acting B cell differentiation factor for antibody-forming cells and germinal center reactions. Overproduction of IL-6 has been reported in several inflammatory autoimmune diseases, including RA, systemic lupus erythematosus, and systemic juvenile idiopathic arthritis (14, 15). Elevated IL-6 levels have also been linked with disease activity and progression in RA (16). TNF- α is a key pathogenic cytokine, playing a central role in RA through the activation of a cytokine cascade driving the inflammation and tissue damage. TNF-α is produced by various cell types, including lymphocytes (B and T cells), monocytes, macrophages, dendritic cells, synovial fibroblasts, mast cells, and natural killer cells (17–19). Along with IL-1β, TNF-α up-regulates RANKL (receptor activator of nuclear factor kappa-B ligand) and promotes osteoclast differentiation and bone resorption (20, 21). Serum levels of TNF-α have been shown to negatively correlate with B cell functions. Furthermore, an increased plasma level of TNF-α can induce TNF-α production from unstimulated B cells without antigenic stimulation, resulting in B cells possessing a pre-activated phenotype which renders them incapable of normal functions (22, 23).

Biological agents targeting key pro-inflammatory cytokines, including IL-6 (24, 25), and TNF-α, have substantially advanced in the treatment of autoimmune disorders (26). Tocilizumab (TCZ) is a humanized recombinant IgG1 monoclonal antibody that binds to the soluble and membrane-bound IL-6 receptor (27, 28). Adalimumab (ADA) is a fully human monoclonal antibody binding TNF, approved for the treatment of RA, either alone or in combination with disease-modifying antirheumatic drugs (DMARDs) demonstrating substantial experience in terms of efficacy and safety (29).

Enhanced B cell activity, particularly memory B cells, has drawn interest in evaluating the therapeutic response to biologics. B cell activation and its modulation through cytokine inhibition therapies have not been thoroughly investigated. Therefore, in this study, we aimed to explore the impact of distinct cytokine inhibitions on the activation status of B cell subpopulations. Hence, we analyzed the activation status and chemokine receptors expressed in different B cell compartments in RA during *in vivo* IL-6R (tocilizumab) and TNF-α (adalimumab) inhibition. Our results suggested that chronic inflammation leads to changes in chemokine receptor expression on peripheral blood B cells. This activation can be successfully modulated using cytokine inhibition therapies.

## Materials and Methods

### Patients

In total, 80 patients with RA, with a median age of 53 years (range 35–73 years), and 40 age-matched healthy donors (HD) were selected in this study. These patients presented with active RA and were regarded as inadequate responders to classical treatment with conventional synthetic DMARD (csDMARD). These patients demonstrated a median disease duration of 8 years (range 2–28), and 73% were female. Patients were considered eligible for study participation if they met the revised 1987 criteria of the American College of Rheumatology (ACR) for RA classification or the 2010 ACR/European League Against Rheumatism (EULAR) classification criteria (30). The study protocol was approved by the ethics committee of the University Hospital, Würzburg, Germany, and was carried out in accordance with the Declaration of Helsinki and Good Clinical Practice. Written informed consent was obtained from all patients. Human material was stored according to standards of the Interdisciplinary Bank of Biomaterials and Data Würzburg at the University of Würzburg (see: www.ibdw.uk-wuerzburg.de). The enrolled patients (*n* = 60) were administered 8 mg/kg TCZ every 4 weeks as a 60-min infusion in combination with methotrexate (MTX). In parallel, for TNF-α inhibition, 20 patients were administered ADA at a dose of 40 mg every 2 weeks in combination with MTX. The primary endpoint was set at 12 weeks, with an extension period to 24 weeks for both TCZ and ADA.

### Clinical Assessments and Evaluation of Efficacy

Demographic and clinical characteristics of patients were regularly monitored by measuring Disease Activity Score 28 (DAS28), RF levels, C-reactive protein (CRP) levels, and erythrocyte sedimentation rate (ESR) values. Before therapy, DAS28 scores for TCZ treated patients were 5.16 ± 1.31 (mean ± SD) with 95% CI (4.75–5.79) and of ADA treated patients were 4.78 ± 0.9 (mean ± SD) with 95% CI (3.99–5.57) before therapy. Furthermore, CRP levels were similar in TCZ treated (0.59 ± 0.09 mg/dl) and ADA treated (0.88 ± 0.4 mg/dl) patients before therapy. Table 1 summarizes the clinical characteristics of patients receiving TCZ and ADA therapy. During treatment, DAS28 declined significantly at week 12 and week 24 (*p* < 0.0001), respectively. After the first infusion, inflammatory parameters, CRP and ESR, declined significantly, and stayed negative throughout the subsequent study period. No serious adverse events or serious infections were observed during the study. Similar effects were observed in patients treated with either TCZ or ADA.

**TABLE 1:** Patients characteristics and clinical evaluation of effectiveness.

	Baseline	Week 12	Week 24
Age, Median (range) years	53 (35–73)		
% of female	73%		
Disease duration, mean (range) years	8 (2–28)		
RF positive	59		
ACPA positive	55		
RF positive/ACPA positive	55		
RF positive/ACPA negative	11		
RF negative/ACPA negative	14		
Concomitant DMARD (patients)	60 TCZ, 20 ADA		
Concomitant Steroid (patients)	36 TCZ, 11 ADA		
Number of Previous DMARD	3 (Methotrexate, Leflunomide, Azathioprine)		
Patients with Rituximab therapy	0		
Patients completed the TCZ study	60	58	53
Patients completed the ADA study	20	20	19

**Tocilizumab (*n* = 60)**	***n* = 60**	***n* = 58**	***n* = 53**

DAS28 score (mean ± SD)	5.16 ± 1.31	3.22 ± 1.31ψ	2.65 ± 1.22ψ
CRP, mg/dl (mean ± SEM)	0.59 ± 0.09	0.14 ± 0.05ψ	0.08 ± 0.03ψ
ESR, mm/hour (mean ± SEM)	29.41 ± 3.15	9.06 ± 1.30ψ	7.11 ± 0.84 ψ
Patient’s VAS. (mean ± SEM)	58.21 ± 4.16	46.97 ± 4.21 ψ	33.21 ± 4.74 ψ

**Adalimumab (*n* = 20)**	***n* = 20**	***n* = 20**	***n* = 19**

DAS28 score (mean ± SD)	4.78 ± 0.9	2.43 ± 0.71 ψ	1.91 ± 0.61 ψ
CRP, mg/dl (mean ± SEM)	0.88 ± 0.4	0.25 ± 0.05ψ	0.12 ± 0.04 ψ
ESR, mm/hour (mean ± SEM)	26.67 ± 6.1	12.86 ± 3.3ψ	8.57 ± 1.7 ψ
Patient’s VAS (mean ± SEM)	57.14 ± 7.3	26.43 ± 7.5 ψ	19.29 ± 2.9 ψ

*The primary endpoint of the study was a reduction in the Disease Activity Score in 28 joints (DAS28) at week 24. Except where indicated, values are the mean ± SEM. RF, rheumatoid factor; ACPA, anti-citrullinated peptide/protein antibody; CRP, C-reactive protein; ESR, erythrocyte sedimentation rate; and VAS, visual analog scale (100-mm). ^Ψ^*P* < 0.05 vs baseline.*

### Flow Cytometry

EDTA anticoagulated peripheral blood was used for phenotype analysis by flow cytometry. In detail, 200 μL of whole blood was lysed in 2 mL of VersaLyse at room temperature for 15 min. Then, cells were washed twice with FACS buffer, followed by resuspension in the appropriate antibody preparation and incubated for 20 min at 4°C. For intracellular staining of Ki-67 expression, we used permeabilization and fixation method using eBioscience perm/fix kit (ebioscience cat no. 88-8824-00). The following monoclonal antihuman antibodies were used in appropriate concentrations to stain cells: anti-CD45-Krome orange (Beckman Coulter, cat no. 96416), anti-CD14-PC5.5 (Beckman Coulter, cat no. A70204), anti-CD 19-APC-Alexa fluor 750 (Beckman Coulter, cat no.A94681), anti-CD27-PE (BD Pharmingen, cat no.555441), anti-CXCR3-APC (BD Pharmingen, cat no. 561732), anti-CXCR4-APC (BD Pharmingen, cat no. 560936), anti-CD95-APC (BD Pharmingen, cat no. 558814), anti-ki-67-PE (Ebioscience Cat no. 12-5699-42), and anti- IgD-FITC (BD Pharmingen, cat no. 555778). After staining, the cells were analyzed by 10-color flow cytometry (Navios, Beckman Coulter), with at least 20,000 CD19+ events collected for each analysis.

### Statistical Analysis

Statistical analysis was performed using GraphPad Prism 7.0 (GraphPad Software, San Diego, CA, United States) and SPSS version 22 (IBM Corp., Armonk, NY, United States). The values were compared with baseline levels using the Mann–Whitney *U* test and the nonparametric Wilcoxon matched-pair test. Univariate logistic regression was performed to calculate the odds ratios and correlated using Pearson’s *r*. All *p*-values ≤ 0.05 were considered statically significant. ***p < 0.0001, **p < 0.001, and *p < 0.01.

## Results

### High Prevalence of Activated B Cells in RA Patients

**FIGURE 1:**
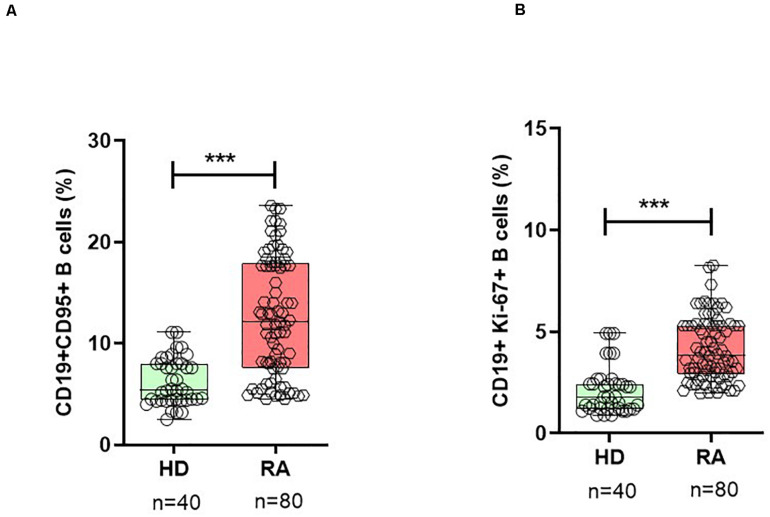
Activated B cells are expanded in RA. In patients with RA patients, B cells show a significantly higher percentage of surface CD95+ **(A)** and intracellular Ki-67+ **(B)** expressing B cells when compared with healthy donors (HD). Data are shown in box-whisker plots showing individual dots, where boxes represent 25th to 75th percentiles and the lines within the boxes represent the median. *P* values were determined with the Student’s unpaired *t*-test using GraphPad Prism 6 (***P < 0.0001). *n* = number of individuals.

To evaluate the activation status and homeostatic proliferation of B cells during active RA, we analyzed the surface expression of CD95 and intracellular Ki-67 expression on B cell subsets. Patients with RA demonstrated a significantly high number of both CD19+CD95+ and CD19+Ki-67+ B cells when compared with HD (Figure 1 and Supplementary Figure 1).

**FIGURE 2:**
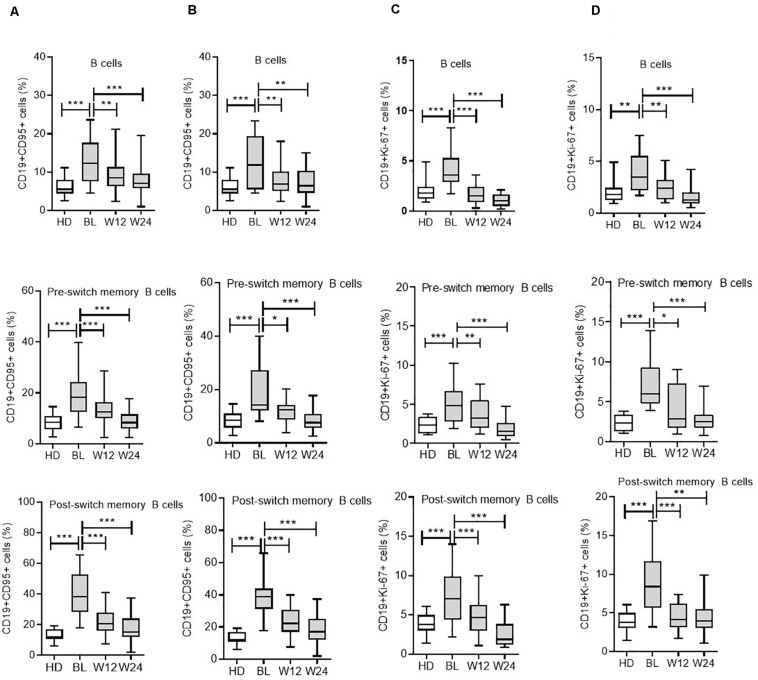
Modulation of CD95 expression on B cell subsets during IL-6R and TNF-α inhibition therapy. For CD19+CD95+ expressing cells from all B cell subsets, including total B cells, pre-switch memory, and post-switch memory B cells, are significantly reduced during IL-6R inhibition **(A)** and TNF-α inhibition **(B)** therapy. CD19+Ki-67+ expressing cells from all B cell subsets are significantly reduced during IL-6R inhibition **(C)** and TNF-α inhibition **(D)** therapy. Values were consistently compared with baseline levels by using the Student unpaired *t*-test (***p < 0.0001, **p < 0.001, and *p < 0.01). BL = baseline, W12 = week 12, and W24 = week 24.

In patients with RA, the mean frequency of CD19+CD95+ on B cells was 12.7 ± 0.6% (mean ± SEM) when compared with 6.3 ± 0.4% in HD (*p* < 0.0001). Based on the surface expression of IgD and CD27, human peripheral CD19+B cells can be divided into four subsets (31): mature naïve B cells (CD19+IgD+CD27-), pre-switch (CD19+IgD+CD27+), post-switch (CD19+IgD-CD27+) conventional, and double-negative (DN; CD19+IgD-CD27-) largely “atypical” memory B cells (Supplementary Figure 1). Analysis of B cell subsets for CD19+CD95+ expressing cells showed that the post-switch subset presented the highest expression of CD95+ cells, followed by DN and pre-switch memory B cells, respectively, in RA patients (Figure 2). Compared with all three memory B cell subsets, the naïve B cell subset demonstrated significantly low CD95 surface expression (Figure 2 and Supplementary Figure 2). In detail, the mean frequency of CD19+CD95+ expressing cells was as follows: in post-switch memory (CD19+IgD-CD27+CD95+) cells (*p* < 0.0001, mean ± SEM in RA = 38.9 ± 1.5% vs. HD = 18.6 ± 1.4%); in pre-switch memory (CD19+IgD+CD27+CD95+) B cells (*p* < 0.0001, RA = 19.7 ± 1.2% vs. HD = 9.2 ± 0.96%); in DN B cells (*p* < 0.0001, RA = 22.1 ± 1.0% vs. HD = 12.0 ± 0.9%); and in naïve B (CD19+IgD+CD27-CD95+) cells (*p* = 0.018, mean ± SEM in RA = 1.3 ± 0.1% vs. HD = 0.86 ± 0.1%).

The analysis of intracellular Ki-67 expression on B cells (CD19+Ki-67+) demonstrated that patients with RA presented significantly higher expression (4.2 ± 0.2) when compared with HD (2.2 ± 0.2; *p* < 0.0001) in HD (Figure 1B). Ki-67 expressing cells were significantly higher in all three memory B cell subsets, except in naïve B cells. The highest Ki-67 expression was observed in post-switch memory (mean 7.8%) B cells (CD19+IgD-CD27+Ki-67+), followed by pre-switch memory (mean 5.3%) B cells, and DN memory B cells (mean 3.8%), respectively, (Figure 2C). The expression of this marker was significantly higher in RA patients when compared with HD. In detail, post-switch memory B cells (*p* = 0.013, mean ± SEM in RA = 7.8 ± 0.6% vs. HD = 4.9 ± 0.7%); pre-switch memory (CD19+IgD+CD27+Ki-67+) B cells (*p* = 0.0002, RA = 5.3 ± 0.5% vs. HD = 2.2 ± 0.3%); DN memory B (CD19+IgD-CD27-Ki-67+) cells (*p* = 0.010, RA = 3.8 ± 0.3% vs. HD = 2.5 ± 0.3%), and in naïve B (CD19+IgD+CD27-Ki-67+) cells (*p* = 0.89, RA = 0.6 ± 0.1% vs. HD = 0.6 ± 0.1%).

### Activated B Cells Positively Correlate With Disease Activity

**FIGURE 3:**
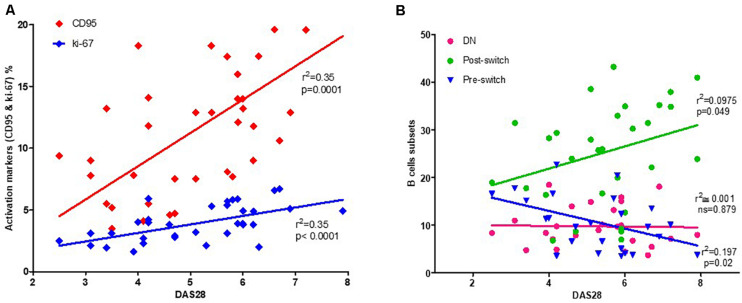
Correlation between activated B cells and disease activity. A significant correlation can be observed between activated B cells, identified by their CD95+ or Ki-67+ expression, and DAS28 as a measure of RA disease activity **(A)**. Among B cell subsets, post-switch exhibits a positive correlation, pre-switch a negative correlation, while double-negative (DN) B cells fail to demonstrate any correlation with DAS28 **(B)**. The relationship between variables was evaluated using the Pearson linear correlation test. A two-sided *p*-value of <0.05 was considered statistically significant.

As activated B cells might reflect disease activity, we correlated CD95+ and Ki-67+ expressing B cells in RA patients. Interestingly, using linear Pearson’s correlation, we observed a positive correlation between the DAS28 score and CD95 expression (*r*^2^ = 0.35, *p* = 0.0001). Similarly, DAS28 was significantly correlated with Ki-67 expression (*r*^2^ = 0.35, *p* = 0.0001; Figure 3A).

As the subset compositions of the B cell compartment may be influenced by disease activity, we assessed the potential correlation between DAS28 and the percentage of each B cell subset. Herein, we observed a positive correlation between DAS28 (Figure 3B) and post-switch memory B cells (*r*^2^ = 0.097, *p* = 0.049). However, pre-switch memory B cells were negatively correlated (*r*^2^ = 0.197, *p* = 0.02), with no correlation observed between DAS28 and DN B cells (*r*^2^ = 0.001, *p* = 0.879). During treatment, non-significant correlation was observed at W12 and W24 (Supplementary Figure 3).

### Modulation of CD95 and Ki-67 Expressions on B Cells Following IL6 Receptor Inhibition Induced by Tocilizumab

Subsequently, we determined whether therapy using an anti-IL-6R inhibitor (TCZ) can modulate CD95+ and Ki-67+ expressing B cells, as well as their subsets. Interestingly, both CD95 and Ki-67 expressions were significantly reduced in the B cell compartment during cytokine inhibition with TCZ (Figure 2C and Supplementary Figure 2). In detail, during TCZ therapy, CD95+ expressing B cells were significantly reduced from 12.5 ± 0.7% (mean ± SEM) to 9.5 ± 0.7% at week 12 (*p* = 0.007), and further reduced to 8.0 ± 0.6% at week 24 (*p* < 0.0001). Additionally, an analysis of B cell subsets demonstrated a significant reduction in CD19+CD95+ expressing cells in all B cell subsets investigated (Figure 2A).

Similarly, Ki-67+ expressing B cells were significantly reduced from 4.1 ± 0.3% (mean ± SEM) to 1.7 ± 0.2% at week 12 (*p* < 0.0001) and 1.0 ± 0.1% at week 24 (<0.0001). This reduction in CD19+Ki-67+ cells following IL-6R inhibition was observed across all B cell subsets (Figure 2C).

### Modulation of CD95 and Ki-67 Expression by B Cells During TNF-α Blockade

To compare the effects of different cytokine inhibition therapies on the activation state of B cells, we analyzed CD19+CD95+ and CD19+Ki-67+ expressing cells during TNF-α blockade using ADA. Similar to TCZ, following ADA therapy, CD95 expression on B cells was significantly reduced (Figure 2B) from 13.1 ± 1.6% (mean ± SEM) to 7.8 ± 0.8% at week 12 (*p* = 0.004) and 7.5 ± 0.8% at week 24 (*p* = 0.002). Furthermore, the B cell subset analysis presented corresponding reduced CD95+ cells (Figure 2B).

During ADA treatment, analysis of CD19+Ki-67+ expressing B cells revealed reduced expression of Ki-67, from 4.0 ± 0.5% (mean ± SEM) to 2.5 ± 0.3% at week 12 (*p* = 0.02) and 1.5 ± 0.3% at week 24 (*p* < 0.0001). Likewise, the intracellular expression of Ki-67+ cells, an indicator of proliferation, was significantly reduced in all B cell subsets during ADA treatment (Figure 2D). These findings indicate that ADA can modulate the activation status of B cells in RA.

### Chemokine Receptor CXCR3 and 4 Expressions by B Cells and Their Modulation Using Biologics

**FIGURE 4:**
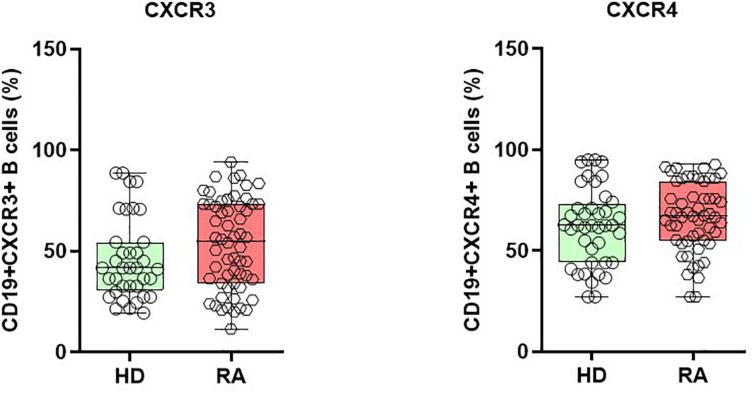
Enhanced expression of chemokine receptor CXCR3 and CXCR4 on B cells during active RA. CD19+CXCR3+ and CD19+CXCR4+ expressing B cells are elevated in patients with active RA when compared with healthy donors (HD).

Under pathological conditions, chemokines are known to direct lymphocytes into inflamed tissues by interacting with chemokine receptors. Therefore, we questioned if, and how, the expression of chemokine receptors, CXCR3, and CXCR4, on B cells is affected in patients with active RA. In both CD19+CXCR3+ and CD19+CXCR4+ chemokine receptors expressing B cells, we observed a non-significant numerical increase in RA patients when compared with HD (Figure 4). In detail, CD19+ CXCR3+ expressing B cells comprised 53.3 ± 2.9% (mean ± SEM) in RA and 47.1 ± 3.7 % in HD (*p* = 0.190), whereas the CD19+CXCR4+ B cells were 66.3 ± 2.5% in RA and 61.3 ± 3.4% in HD (*p* = 0.24). Among the different B cell subsets, naïve B (CD19+IgD+CD27-) cells numerically presented the highest percentage of CD19+CXCR3+, as well as CD19+CXCR4+ cells, followed by pre-switch memory B cells, DN B cells, and post-switch memory B cells, respectively.

**FIGURE 5:**
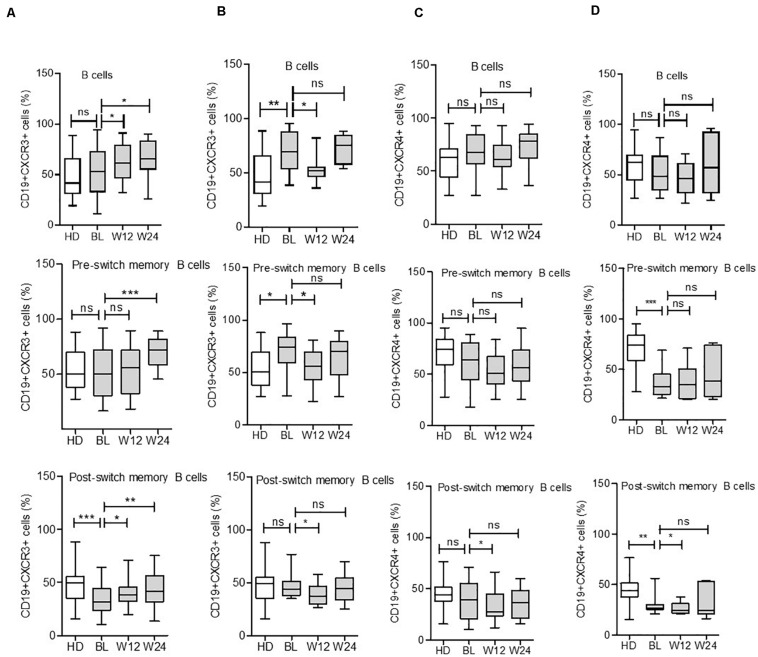
Expression of CXCR3 and CXCR4 in B cell subsets during IL-6R and TNF inhibition therapy. CXCR3 expression on B cell subsets increases in some subsets during IL-6R inhibition **(A)** and decreases during TNF-α inhibition **(B)** therapy. CXCR4 expression on B cell subsets reduces in certain subsets during IL-6R inhibition **(C)** and TNF-α inhibition **(D)** therapy. Values were consistently compared with baseline levels using the Student unpaired *t*-test (***p < 0.0001, **p < 0.001, and *p < 0.01). BL = baseline, W12 = week 12, and W24 = week 24.

Interestingly, we observed significant alternations in CXCR3 chemokine receptors expressing B cells during cytokine inhibition therapies. In detail, during TCZ treatment, the mean CD19+CXCR3+ expressing cells increased from a baseline value of 52.3 ± 3.1 to 63.2 ± 2.7% (*p* = 0.010) at week 12 and 65.3 ± 3.6% (*p* = 0.011) at week 24. A similar pattern was observed in all B cell subsets during TCZ treatment (Figure 5A and Supplementary Figure 4). Similarly, during ADA treatment, the expression pattern of CD19+CXCR3+ B cells reduced from 68.2 ± 4.6% at baseline to 54.2 ± 3.3% at week 12 (*p* = 0.019), with subsequent elevation to 72.2 ± 3.1% (*p* = 0.47) at week 24 for total B cells. Similar significant changes were observed in the expression pattern of CD19+CXCR3+ cells at week 12; these changes were non-significant at week 24 for all B cell subsets (Figure 5B).

Contrary to CXCR3 modulation during cytokine inhibition therapies, CD19+CXCR4+ receptor expressing B cells presented a reduced or unchanged pattern following TCZ or ADA treatment. Among B cell subsets, the CD19+CXCR4+ expressing cells were significantly decreased in post-switch memory B cells during both treatments, with a significant decrease observed in DN B cells after ADA treatment at week 12. However, these differences were mild at week 24 (Figures 5C,D and Supplementary Figure 4). These findings suggested that the CD19+CXCR3+ and CD19+CXCR4+ expressing B cells present distinct patterns during active RA disease, which were differentially modulated during cytokine inhibition therapies.

## Discussion

The use of monoclonal antibodies against cytokines has opened new therapeutic modalities for patients with RA. Currently, specific biologics such as TNF-α inhibitors and IL-6 receptor antibodies are considered highly efficacious therapeutic agents in RA treatment. Immune cell monitoring may help assess these therapies to reach optimal responses (3, 32, 33). Therefore, the main objective of this study was to explore the impact of *in vivo* IL-6 or TNF-α inhibition on the activation status of B cells. Hence, we analyzed activation status and chemokine receptor expressions in different B cell subsets from RA patients during IL-6R (TCZ) and TNF-α (ADA) inhibition. Previous data from experimental arthritis have shown a gender-based differences in B cell signatures (34, 35), however, in our cohort, we did not observe any gender-biased variation in B cell subsets (data not shown). We longitudinally analyzed patients undergoing treatment with these biologics for 24 weeks. Additionally, we analyzed B cell activation and proliferation based on surface expression of CD95 and intracellular expression of Ki-67, extending our investigation to different B cell subsets and their modulation during cytokine inhibition. Our data revealed an activated phenotype in B cells, particularly in memory B cell subsets, including DN, pre-switch, and post-switch B cells in RA patients (*n* = 80) when compared with HD (*n* = 40) at baseline (Figures 1, 2). Compared with HD, these activation and proliferation markers of B cells (CD95 and Ki-67) were significantly higher in RA patients (*p* < 0.0001). Our data did not reveal any differences in B cell activation status in patients treated with TCZ or ADA along with either concomitant DMARD or concomitant steroid. Elevated B cell activation has been reported in autoimmune diseases like SLE and RA, where B cells are considered to play a role in pathogenesis (36, 37). Reportedly, a previous report has demonstrated that the expressions of CD95 and CD86 were up-regulated in the B cells of new-onset RA patients and positively correlated with DAS28 (37). Similar reports in SLE have shown that the CD95 expression on B cells was increased in relation to their activation, and correlated with disease activity (38). Reportedly, SLE patients present higher CD95 expression in CD27+ B cells (39), and CD27-IgD- CD95+B cells were shown to be associated with active disease (40). In our patient cohort, disease activity, as determined by DAS28 and other inflammatory factors like ESR and CRP levels, was elevated. We observed a positive correlation between DAS28 and B cells expressing CD95 and Ki-67 (Figure 3). Under cytokine inhibition therapies using anti-IL6R and anti-TNF-α, the higher activity of total B cells and their subsets expressing CD95 and Ki-67 was significantly reduced, along with DAS28 score and other inflammatory factors (ESR, CRP levels; Table 1). In B cells, we observed a significant decline in the expression of surface CD95 and intracellular Ki-67 during TCZ (<0.0001) and ADA (<0.001) therapy. During both cytokine inhibition therapies, CD95 and Ki-67 expressions in all B cell memory subsets investigated in the present study were significantly reduced from baseline to week 12 and week 24 (Figure 2).

Among the B cell subsets, the post-switch memory B cells demonstrated the highest expressions of CD95 and Ki-67, followed by pre-switch and DN memory B cells. This implies that the overall B cell activation might largely depend on post-switch B cells (Figure 2). Previous reports have indicated that anti-TNF treatment influences the expression of the costimulatory molecule, CD86, as well as that of the inhibitory receptor, Fcγ receptor IIb (FcγRIIb) (41), on B cells. Furthermore, our data corroborate with previous reports showing a decreased expression of activation marker CD69, along with an increase in regulatory B cells after ADA (42) and TCZ treatments (14), indicating that cytokine inhibition therapy can normalize peripheral B cells. In this regard, recent functional investigations of RA, SLE, and Sjögren’s B cells have identified that particularly memory B cells from patients in an anergic or post-activated status demonstrate reduced B cell receptor responsiveness and cytokine production (43). Most noteworthy and consistent with the potential reversal of these functional impairments, TCZ treatment reportedly improves the capacity of cytokine production by B cells in RA (44). These findings suggest that anti-cytokine therapy in patients with RA results in detectable improvements of B cell functions, thus supporting the current findings.

Chemokines regulate cellular migration to various physiological and pathological processes via chemokine receptors, contributing to B cell migration, as well as their proliferation and cytokine production in RA (36). CXCR3 is a chemokine receptor for inflammatory chemokines, including CXCL9, 10, and 11 (45). Higher levels of CXCR3 expressing T cells have been documented in patients with RA and correlate with disease activity (46). CXCR3 knockout mice are reportedly resistant to inflammatory autoimmune disease (47). Compared to HD, we observed a numeric increase in CD19+CXCR3+ expressing B cells in RA patients (Figure 4). Interestingly, CD19+CXCR3+ expressing B cells were present in naïve, as well as memory B cell subsets (Figure 5). During TCZ treatment, the B cell subset expression of CXCR3 increased, particularly in the post-switch memory compartment; however, during ADA therapy, CXCR3 expressing cells decreased significantly during the first 12 weeks and remained unaltered in later weeks. This data may indicate that cytokine therapies demonstrate differential effects on B cell subsets. As IFN-γ is involved in the induction of CXCR3 expression on B cells (45), we postulate that different cytokine inhibitions induce differential T cell responses, and IL-6R inhibition by TCZ might lead to Th1-biased immunomodulation. Interestingly, IFN-γ producing Th1 cells are elevated in RA patients during TCZ therapy (48), whereas a decrease in Th1 cells has been documented during ADA treatment (49). Chemokine receptor CXCR4+ B cells demonstrated a higher expression tendency in RA patients when compared with HD (Figure 4B). CXCR4 is expressed by B cell subsets throughout the B cell ontogeny and its ligands. CXCL12 is broadly distributed in various tissues (50). CXCR4 is an essential homing receptor and is required for the normal accumulation of plasma cells, as well as for retaining developing B cells in the bone marrow (45, 51). Additionally, CXCR4 and its ligand, CXCL12, are reportedly involved in the pathogenesis of RA (52). Our data showed a marginal reduction in CD19+CXCR4+ B cells during week 12 of treatment; however, this modulation was not statistically significant. In both TCZ and ADA treatment, this reduction was not observed during week 24, indicating the minimal influence of anti-cytokine therapy on CD19+CXCR4+ B cells (Figure 5). A detailed analysis of B cell subsets for the expression of CXCR4 demonstrated the significant modulation on CD19+CXCR4+ post-switch memory B cells.

In conclusion, our study revealed a phenotype of activated B cell subsets, particularly in the post-switch B cell compartment, in patients with active RA, and its successful modulation during cytokine inhibition therapies. The higher expressions of surface CD95 and intracellular Ki-67 at baseline reflected the disease activity and are positively correlated with RA disease activity. The subtle change observed in the expressions of chemokine receptors, CXCR3 and CXCR4, indicates that the migratory and homing capacity of B cells might be altered during cytokine inhibition. Our data further contribute to knowledge regarding the therapeutic effects of cytokine inhibitors on peripheral B cells. The B cell activation status may be further explored as predictors of response during such therapies.

## Data Availability Statement

The raw data supporting the conclusions of this article will be made available by the authors, without undue reservation.

## Ethics Statement

The studies involving human participants were reviewed and approved by the ethics committee of the University Hospital, Würzburg, Germany and was carried out in accordance with the Declaration of Helsinki and Good Clinical Practice. The patients/participants provided their written informed consent to participate in this study.

## Author Contributions

ZM, H-PT, and KM conceptualized the project. ZM, MS, and KM were involved in data acquisition and analysis. MS was involved in clinical studies and revising the manuscript. TD provided reagents and critically revised the manuscript. KM led the investigation and wrote the manuscript with the help of ZM and H-PT. All authors approved the final version to be published. H-PT and KM had full access to all of the data in the study and take responsibility for the integrity of the data and the accuracy of the data analysis.

## Conflict of Interest

The authors declare that the research was conducted in the absence of any commercial or financial relationships that could be construed as a potential conflict of interest. The handling editor declared a shared affiliation, though no other collaboration, with one of the authors, TD.

## References

[1] Vivar N Van Vollenhoven RF. Advances in the treatment of rheumatoid arthritis. *F1000Prime Rep.* (2014) 6:31. 10.12703/P6-31 24860653 PMC4017904

[2] Pitzalis C. Pathogenesis of rheumatoid arthritis: from systemic autoimmunity to localised joint disease. *Drug Discov Today.* (2014) 19:1152–4. 10.1016/j.drudis.2014.05.009 24859017

[3] Elemam NM Hannawi S Maghazachi AA. Role of chemokines and chemokine receptors in rheumatoid arthritis. *Immunotargets Ther.* (2020) 9:43–56. 10.2147/ITT.S243636 32211348 PMC7074856

[4] Klareskog L Stolt P Lundberg K Kallberg H Bengtsson C Grunewald J A new model for an etiology of rheumatoid arthritis: smoking may trigger HLA-DR (shared epitope)-restricted immune reactions to autoantigens modified by citrullination. *Arthritis Rheum.* (2006) 54:38–46. 10.1002/art.21575 16385494

[5] Luque Ramos A Redeker I Hoffmann F Callhoff J Zink A Albrecht K. Comorbidities in patients with rheumatoid arthritis and their association with patient-reported outcomes: results of claims data linked to questionnaire survey. *J Rheumatol.* (2019) 46:564–71. 10.3899/jrheum.180668 30647170

[6] Brennan FM McInnes IB. Evidence that cytokines play a role in rheumatoid arthritis. *J Clin Invest.* (2008) 118:3537–45. 10.1172/JCI36389 18982160 PMC2575731

[7] Rantapaa-Dahlqvist S de Jong BA Berglin E Hallmans G Wadell G Stenlund H Antibodies against cyclic citrullinated peptide and IgA rheumatoid factor predict the development of rheumatoid arthritis. *Arthritis Rheum.* (2003) 48:2741–9. 10.1002/art.11223 14558078

[8] Fekete A Soos L Szekanecz Z Szabo Z Szodoray P Barath S Disturbances in B- and T-cell homeostasis in rheumatoid arthritis: suggested relationships with antigen-driven immune responses. *J Autoimmun.* (2007) 29:154–63. 10.1016/j.jaut.2007.07.002 17826949

[9] Lindenau S Scholze S Odendahl M Dorner T Radbruch A Burmester GR Aberrant activation of B cells in patients with rheumatoid arthritis. *Ann N Y Acad Sci.* (2003) 987:246–8. 10.1111/j.1749-6632.2003.tb06055.x 12727646

[10] Roll P Mahmood Z Muhammad K Feuchtenberger M Dorner T Tony HP. Long-term repopulation of peripheral B-cell subsets after single and repeated rituximab infusions in patients with rheumatoid arthritis. *Clin Exp Rheumatol.* (2015) 33:347–53. 25897997

[11] Martin F Chan AC. Pathogenic roles of B cells in human autoimmunity; insights from the clinic. *Immunity.* (2004) 20:517–27. 10.1016/S1074-7613(04)00112-8 15142521

[12] Barnas JL Looney RJ Anolik JH. B cell targeted therapies in autoimmune disease. *Curr Opin Immunol.* (2019) 61:92–9. 10.1016/j.coi.2019.09.004 31733607 PMC6982404

[13] Barr TA Shen P Brown S Lampropoulou V Roch T Lawrie S B cell depletion therapy ameliorates autoimmune disease through ablation of IL-6-producing B cells. *J Exp Med.* (2012) 209:1001–10. 10.1084/jem.20111675 22547654 PMC3348102

[14] Snir A Kessel A Haj T Rosner I Slobodin G Toubi E. Anti-IL-6 receptor antibody (tocilizumab): a B cell targeting therapy. *Clin Exp Rheumatol.* (2011) 29:697–700. 10.1136/ard.2010.149005.1 21813064

[15] Narazaki M Tanaka T Kishimoto T. The role and therapeutic targeting of IL-6 in rheumatoid arthritis. *Expert Rev Clin Immunol.* (2017) 13:535–51. 10.1080/1744666X.2017.1295850 28494214

[16] Yoshida Y Tanaka T. Interleukin 6 and rheumatoid arthritis. *Biomed Res Int.* (2014) 2014:698313. 10.1155/2014/698313 24524085 PMC3913495

[17] Palladino MA Bahjat FR Theodorakis EA Moldawer LL. Anti-TNF-alpha therapies: the next generation. *Nat Rev Drug Discov.* (2003) 2:736–46. 10.1038/nrd1175 12951580

[18] Gran F Kerstan A Serfling E Goebeler M Muhammad K. Current developments in the immunology of psoriasis. *Yale J Biol Med.* (2020) 93:97–110. 32226340 PMC7087066

[19] deLuca LS Gommerman JL. Fine-tuning of dendritic cell biology by the TNF superfamily. *Nat Rev Immunol.* (2012) 12:339–51. 10.1038/nri3193 22487654

[20] Choy EH Panayi GS. Cytokine pathways and joint inflammation in rheumatoid arthritis. *N Engl J Med.* (2001) 344:907–16. 10.1056/NEJM200103223441207 11259725

[21] McInnes IB Schett G. The pathogenesis of rheumatoid arthritis. *N Engl J Med.* (2011) 365:2205–19. 10.1056/NEJMra1004965 22150039

[22] Pala O Diaz A Blomberg BB Frasca D. B lymphocytes in rheumatoid arthritis and the effects of anti-TNF-alpha agents on B lymphocytes: a review of the literature. *Clin Ther.* (2018) 40:1034–45. 10.1016/j.clinthera.2018.04.016 29801753 PMC6792291

[23] Frasca D Diaz A Romero M Landin AM Blomberg BB. High TNF-alpha levels in resting B cells negatively correlate with their response. *Exp Gerontol.* (2014) 54:116–22. 10.1016/j.exger.2014.01.004 24440385 PMC3989457

[24] Voulgari PV Drosos AA. Adalimumab in the treatment of rheumatoid arthritis. *Expert Opin Biol Ther.* (2014) 14:549–561. 10.1517/14712598.2014.894503 24588123

[25] Choy EH Benedetti F. De Takeuchi T Hashizume M John MR Kishimoto T. Translating IL-6 biology into effective treatments. *Nat Rev Rheumatol.* (2020) 16:335–45. 10.1038/s41584-020-0419-z 32327746 PMC7178926

[26] Carbone G Wilson A Diehl SA Bunn J Cooper SM Rincon M. Interleukin-6 receptor blockade selectively reduces IL-21 production by CD4 T cells and IgG4 autoantibodies in rheumatoid arthritis. *Int J Biol Sci.* (2013) 9:279–88. 10.7150/ijbs.5996 23493630 PMC3596713

[27] Emery P Keystone E Tony HP Cantagrel A van Vollenhoven R Sanchez A IL-6 receptor inhibition with tocilizumab improves treatment outcomes in patients with rheumatoid arthritis refractory to anti-tumour necrosis factor biologicals: results from a 24-week multicentre randomised placebo-controlled trial. *Ann Rheum Dis.* (2008) 67:1516–23. 10.1136/ard.2008.092932 18625622 PMC3811149

[28] Roll P Muhammad K Schumann M Kleinert S Einsele H Dorner T In vivo effects of the anti-interleukin-6 receptor inhibitor tocilizumab on the B cell compartment. *Arthritis Rheum.* (2011) 63:1255–64. 10.1002/art.30242 21305508

[29] Chen YF Jobanputra P Barton P Jowett S Bryan S Clark W A systematic review of the effectiveness of adalimumab, etanercept and infliximab for the treatment of rheumatoid arthritis in adults and an economic evaluation of their cost-effectiveness. *Health Technol Assess.* (2006) 10:iii–iv, xi–xiii, 1–229. 10.3310/hta10420 17049139

[30] Aletaha D Neogi T Silman AJ Funovits J Felson DT Bingham CO III 2010 rheumatoid arthritis classification criteria: an American College of Rheumatology/European League Against Rheumatism collaborative initiative. *Ann Rheum Dis.* (2010) 69:1580–8. 20699241 10.1136/ard.2010.138461

[31] Mahmood Z Muhammad K Schmalzing M Roll P Dorner T Tony HP. CD27-IgD- memory B cells are modulated by in vivo interleukin-6 receptor (IL-6R) blockade in rheumatoid arthritis. *Arthritis Res Ther.* (2015) 17:61. 10.1186/s13075-015-0580-y 25888920 PMC4415279

[32] Alam J Jantan I Bukhari SNA. Rheumatoid arthritis: Recent advances on its etiology, role of cytokines and pharmacotherapy. *Biomed Pharmacother.* (2017) 92:615–33. 10.1016/j.biopha.2017.05.055 28582758

[33] Smolen JS Landewe RBM Bijlsma JWJ Burmester GR Dougados M Kerschbaumer A EULAR recommendations for the management of rheumatoid arthritis with synthetic and biological disease-modifying antirheumatic drugs: 2019 update. *Ann Rheum Dis.* (2020) 79:685–99. 31969328 10.1136/annrheumdis-2019-216655

[34] Dimitrijevic M Arsenovic-Ranin N Kosec D Bufan B Nacka-Aleksic M Pilipovic I Sex differences in Tfh cell help to B cells contribute to sexual dimorphism in severity of rat collagen-induced arthritis. *Sci Rep.* (2020) 10:1214. 10.1038/s41598-020-58127-y 31988383 PMC6985112

[35] Behrens M Luckey D Luthra H David C Taneja V. B cells influence sex specificity of arthritis via myeloid suppressors and chemokines in humanized mice. *Clin Immunol.* (2017) 178:10–9. 10.1016/j.clim.2015.05.015 26057130

[36] Henneken M Dorner T Burmester GR Berek C. Differential expression of chemokine receptors on peripheral blood B cells from patients with rheumatoid arthritis and systemic lupus erythematosus. *Arthritis Res Ther.* (2005) 7:R1001–13. 10.1186/ar1776 16207316 PMC1257429

[37] Wang J Shan Y Jiang Z Feng J Li C Ma L High frequencies of activated B cells and T follicular helper cells are correlated with disease activity in patients with new-onset rheumatoid arthritis. *Clin Exp Immunol.* (2013) 174:212–20. 10.1111/cei.12162 23786438 PMC3828824

[38] Bijl M Horst G Limburg PC Kallenberg CG. Fas expression on peripheral blood lymphocytes in systemic lupus erythematosus (SLE): relation to lymphocyte activation and disease activity. *Lupus.* (2001) 10:866–72. 10.1191/096120301701548517 11787876

[39] Odendahl M Jacobi A Hansen A Feist E Hiepe F Burmester GR Disturbed peripheral B lymphocyte homeostasis in systemic lupus erythematosus. *J Immunol.* (2000) 165:5970–9. 10.4049/jimmunol.165.10.5970 11067960

[40] Jacobi AM Reiter K Mackay M Aranow C Hiepe F Radbruch A Activated memory B cell subsets correlate with disease activity in systemic lupus erythematosus: delineation by expression of CD27, IgD, and CD95. *Arthritis Rheum.* (2008) 58:1762–73. 10.1002/art.23498 18512812

[41] Catalan D Aravena O Sabugo F Wurmann P Soto L Kalergis AM B cells from rheumatoid arthritis patients show important alterations in the expression of CD86 and FcgammaRIIb, which are modulated by anti-tumor necrosis factor therapy. *Arthritis Res Ther.* (2010) 12:R68. 10.1186/ar2985 20398308 PMC2888223

[42] Banko Z Pozsgay J Gati T Rojkovich B Ujfalussy I Sarmay G. Regulatory B cells in rheumatoid arthritis: alterations in patients receiving anti-TNF therapy. *Clin Immunol.* (2017) 184:63–9. 10.1016/j.clim.2017.05.012 28506920

[43] Weissenberg SY Szelinski F Schrezenmeier E Stefanski AL Wiedemann A Rincon-Arevalo H Identification and characterization of post-activated B cells in systemic autoimmune diseases. *Front Immunol.* (2019) 10:2136. 10.3389/fimmu.2019.02136 31616406 PMC6768969

[44] Fleischer S Ries S Shen P Lheritier A Cazals F Burmester GR Anti-interleukin-6 signalling therapy rebalances the disrupted cytokine production of B cells from patients with active rheumatoid arthritis. *Eur J Immunol.* (2018) 48:194–203. 10.1002/eji.201747191 28850672

[45] Muehlinghaus G Cigliano L Huehn S Peddinghaus A Leyendeckers H Hauser AE Regulation of CXCR3 and CXCR4 expression during terminal differentiation of memory B cells into plasma cells. *Blood.* (2005) 105:3965–71. 10.1182/blood-2004-08-2992 15687242

[46] Motoki Y Tani K Shimizu T Tamiya H Hase K Ohmoto Y The expression of chemokine receptor CXCR3: relevance to disease activity of rheumatoid arthritis. *Mod Rheumatol.* (2003) 13:114–20. 10.3109/s10165-002-0209-2 24387169

[47] Karin N Razon H. Chemokines beyond chemo-attraction: CXCL10 and its significant role in cancer and autoimmunity. *Cytokine.* (2018) 109:24–8. 10.1016/j.cyto.2018.02.012 29449068

[48] Pesce B Soto L Sabugo F Wurmann P Cuchacovich M Lopez MN Effect of interleukin-6 receptor blockade on the balance between regulatory T cells and T helper type 17 cells in rheumatoid arthritis patients. *Clin Exp Immunol.* (2013) 171:237–42. 10.1111/cei.12017 23379428 PMC3569529

[49] Aravena O Pesce B Soto L Orrego N Sabugo F Wurmann P Anti-TNF therapy in patients with rheumatoid arthritis decreases Th1 and Th17 cell populations and expands IFN-gamma-producing NK cell and regulatory T cell subsets. *Immunobiology.* (2011) 216:1256–63. 10.1016/j.imbio.2011.07.006 21840621

[50] Nie Y Waite J Brewer F Sunshine MJ Littman DR Zou YR. The role of CXCR4 in maintaining peripheral B cell compartments and humoral immunity. *J Exp Med.* (2004) 200:1145–56. 10.1084/jem.20041185 15520246 PMC2211858

[51] Becker M Hobeika E Jumaa H Reth M Maity PC. CXCR4 signaling and function require the expression of the IgD-class B-cell antigen receptor. *Proc Natl Acad Sci USA.* (2017) 114:5231–6. 10.1073/pnas.1621512114 28461496 PMC5441763

[52] Firestein GS. Evolving concepts of rheumatoid arthritis. *Nature.* (2003) 423:356–61. 10.1038/nature01661 12748655

