# Supplementary material for: Therapeutic Cytokine Inhibition Modulates Activation and Homing Receptors of Peripheral Memory B Cell Subsets in Rheumatoid Arthritis Patients
Source: Front Immunol. 2020 Sep 11;11:572475. doi: 10.3389/fimmu.2020.572475 (PMC7518039; doi:10.3389/fimmu.2020.572475)
Supplement: Supplementary file 1 [file Data_Sheet_1.PDF]

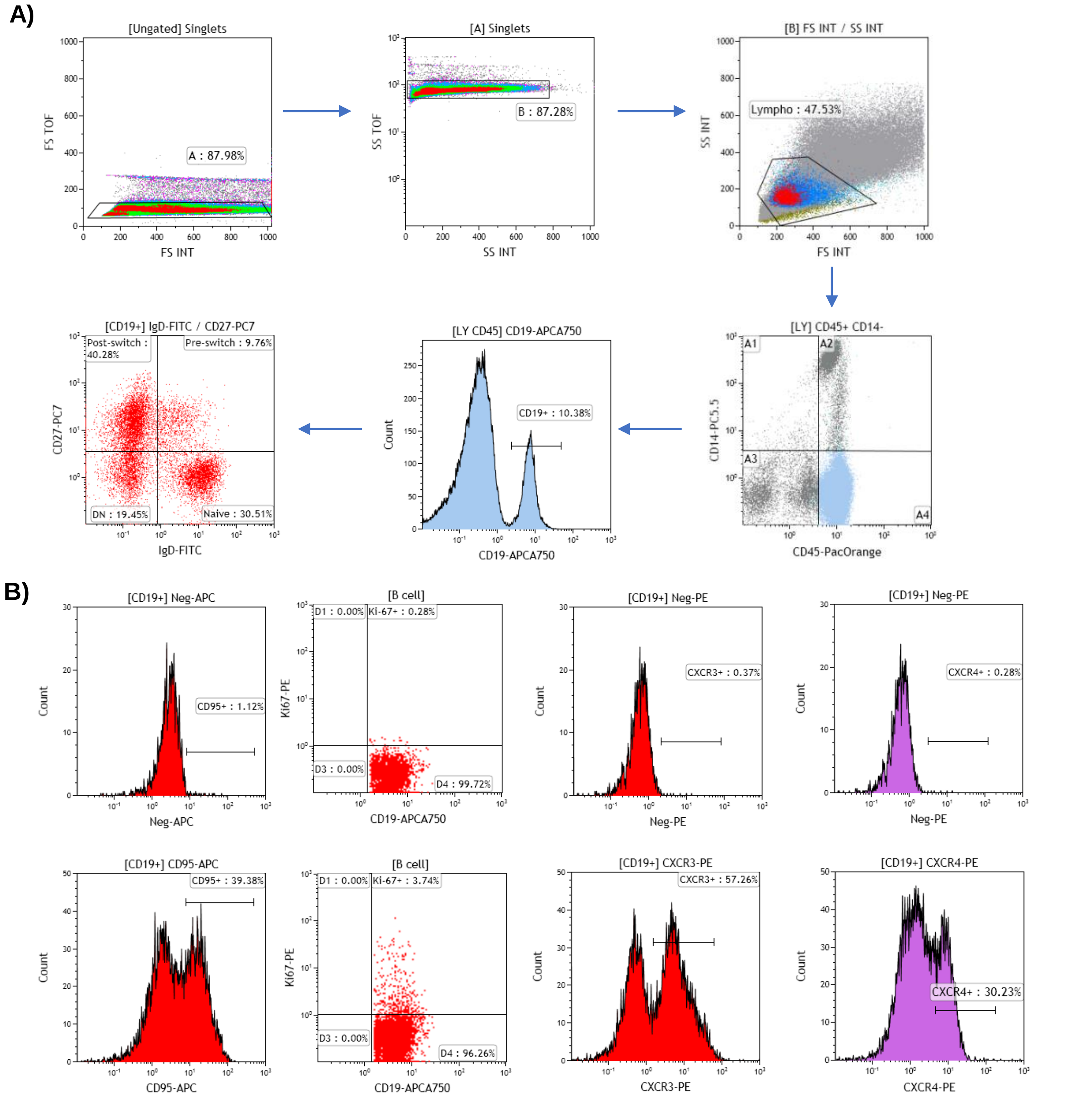

Supplementary Figure 1: Gating strategy of B cell subset identification (A). Representative FACS plots of CD95, Ki-67, CXCR3 and CXCR4 expression by B cells (B).

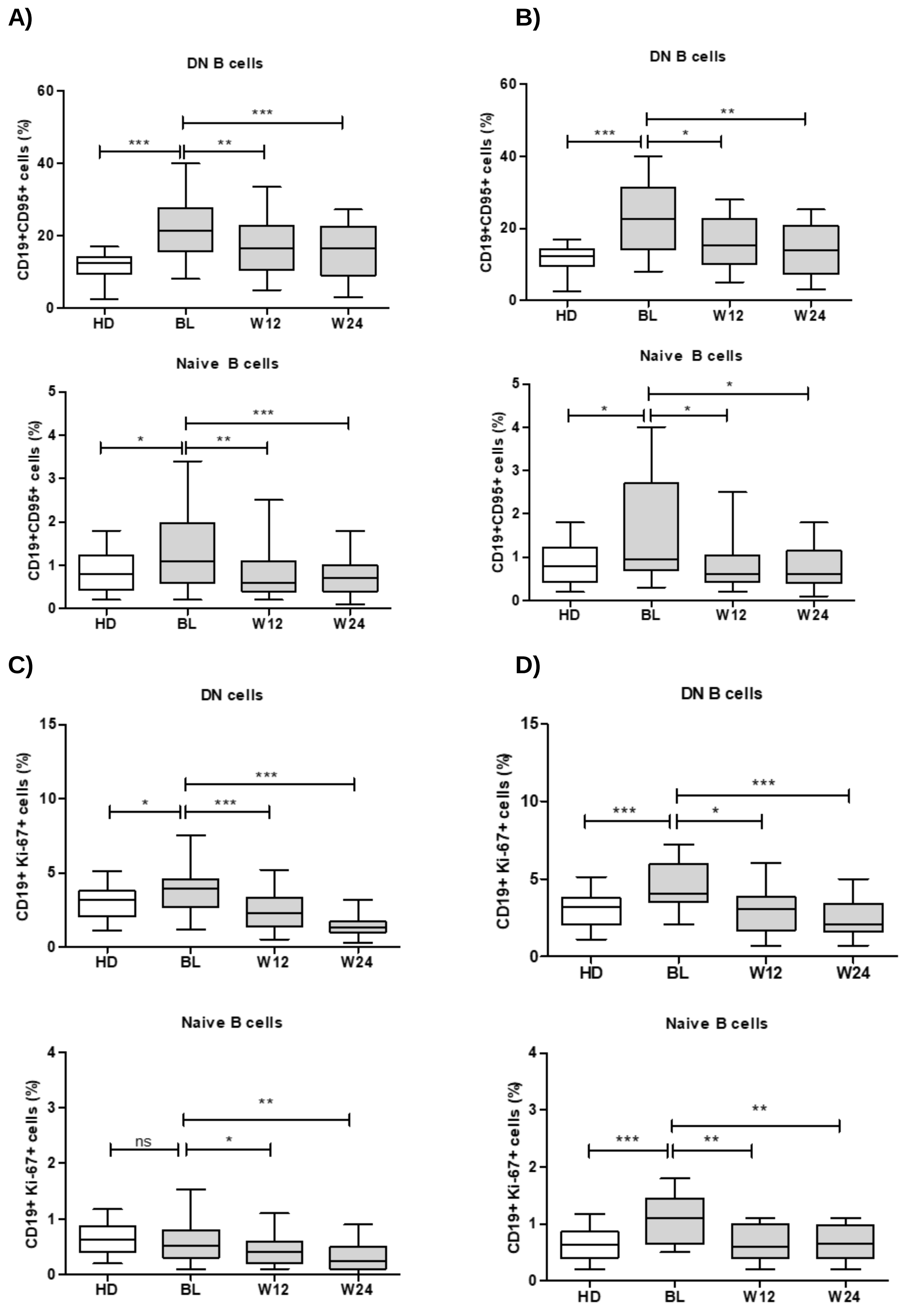

Supplementary Figure 2: Modulation of CD95 expression on B cell subsets during IL-6R and TNF-a inhibition therapy. For CD19+CD95+ expressing cells DN and naïve B cells are significantly reduced during IL-6R inhibition (A) and TNF-a inhibition (B) therapy. CD19+Ki-67+ expressing cells from DN and naïve B cells are significantly reduced during IL-6R inhibition (A) and TNF-a inhibition (B) therapy. Values were consistently compared with baseline levels by using the Student unpaired t-test (***p<0.0001, **p<0.001 and *p<0.01). BL = baseline, W12 = week 12 and W24 = week 24.

A) B)

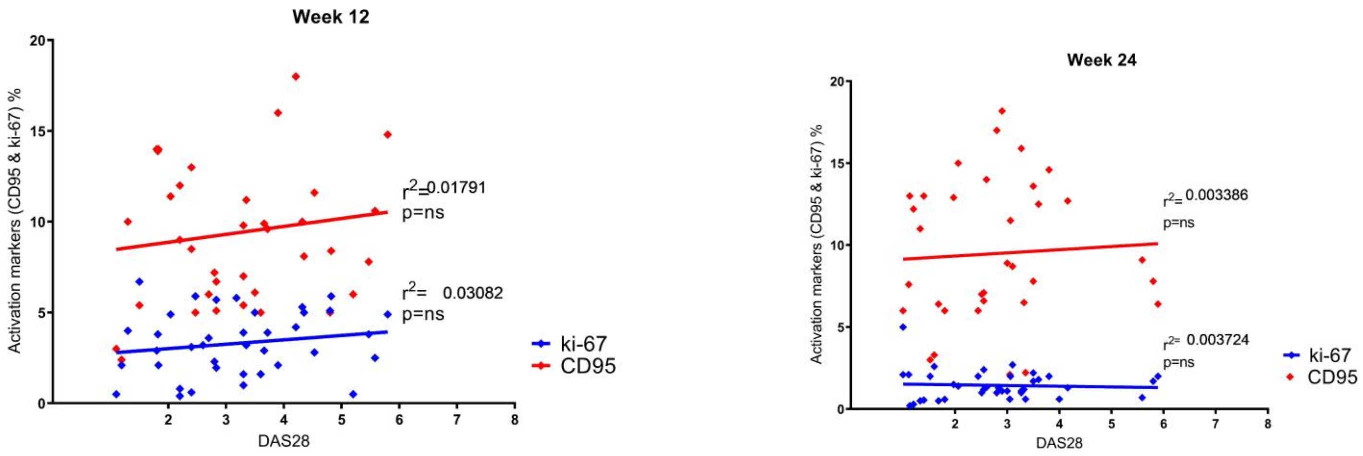

Supplementary Figure 3: Correlation between activated B cells and disease activity during treatment. A non-significant correlation between activated B cells, identified by their CD95+ or Ki-67+ expression, and DAS28 can be observed at week 12 (A) and week 24(B) during cytokine inbition therapies. The relationship between variables was evaluated using the Pearson linear correlation test. A two-sided p-value of <0.05 was considered statistically significant

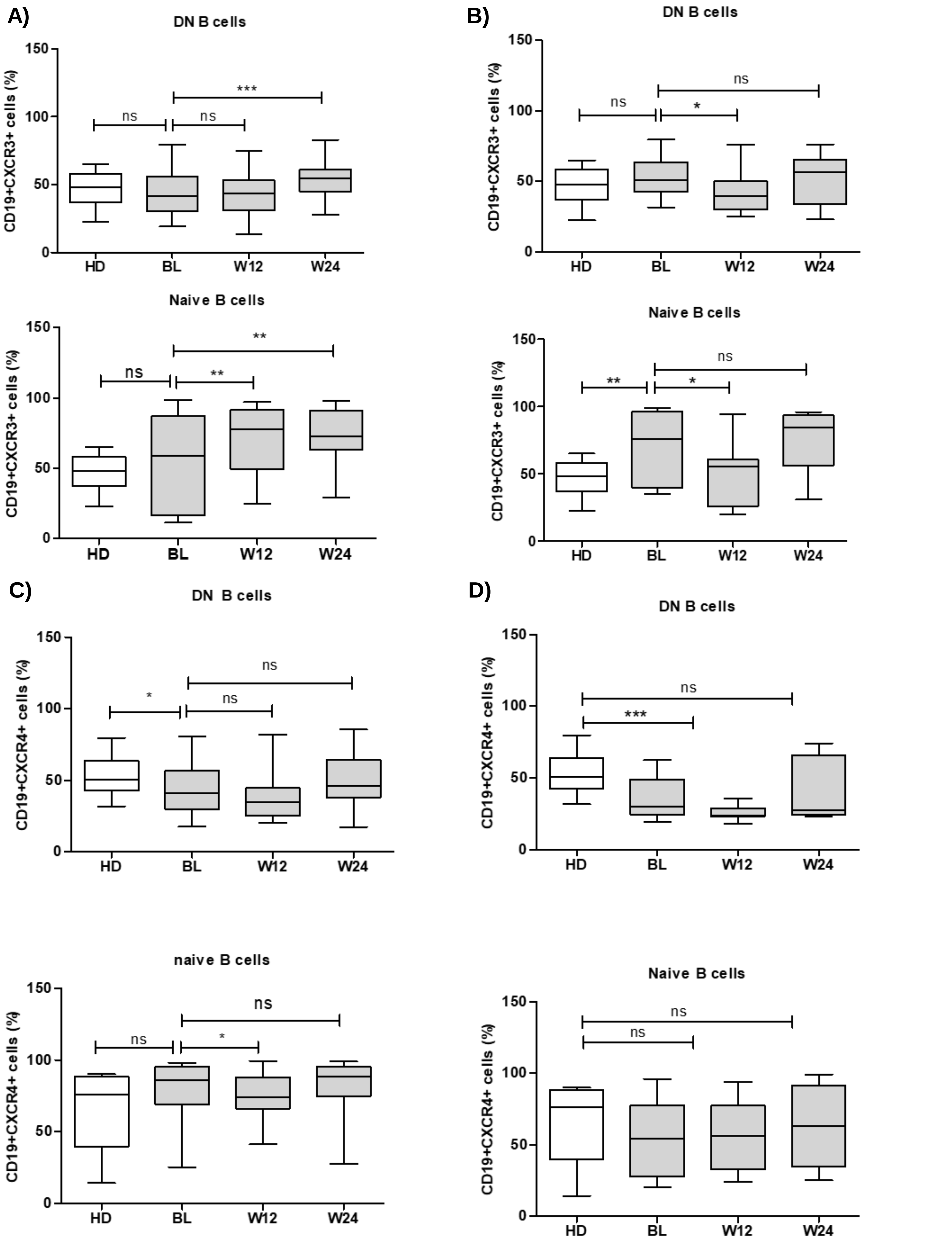

Supplementary Figure 4: Expression of CXCR3 and CXCR4 in B cell subsets during IL-6R and TNF inhibition therapy. CXCR3 expression on DN and naïve B cells during IL-6R inhibition (A) and during TNF-α inhibition (B) therapy. CXCR4 expression on DN and naïve B cells during IL-6R inhibition (C) and TNF-α inhibition (D) therapy. Values were consistently compared with baseline levels using the Student unpaired t-test (***p<0.0001, **p<0.001 and *p<0.01). BL = baseline, W12 = week 12, and W24 = week 24.
